# Global lessons on delivery of primary healthcare services for people with non-communicable diseases: convergent mixed methods

**DOI:** 10.1136/fmch-2023-002553

**Published:** 2024-08-03

**Authors:** Robert Mash, Lisa R Hirschhorn, Inayat Singh Kakar, Renu John, Manushi Sharma, Devarsetty Praveen

**Affiliations:** 1 Stellenbosch University, Stellenbosch, South Africa; 2 Northwestern University, Chicago, Illinois, USA; 3 The George Institute for Global Health, New Delhi, India; 4 The George Institute for Global Health, Hyderabad, India; 5 Manipal Academy of Higher Education, Manipal, India

**Keywords:** Primary Health Care, Health Services, Public Health Systems Research, Chronic Disease

## Abstract

**Objective:**

To extract key lessons on primary healthcare (PHC) service delivery strategies for non-communicable diseases (NCD) from the work of researchers funded by the Global Alliance for Chronic Diseases (GACD).

**Design:**

A convergent mixed methods study that extracted data using a standardised template from research projects funded by the GACD that focused on PHC. The strategies implemented in these studies were mapped onto the PHC Performance Initiative framework. Semistructured qualitative interviews were conducted with researchers from purposefully selected projects to understand the strategies and contextual factors in more depth.

**Setting:**

PHC contexts from low or middle-income countries (LMIC) as well as vulnerable groups within high-income countries. Projects came from all regions of the world, particularly East Asia and Pacific, sub-Saharan Africa, South Asia, Latin America and Caribbean.

**Participants:**

The study extracted data on 84 research projects and interviewed researchers from 16 research projects.

**Results:**

Research projects came from all regions of the world, and mainly focused on diabetes (35.3%), hypertension (28.3%) and mental health (27.6%). Mapped onto the PHC Performance Initiative framework: 49.4% focused on high-quality PHC (particularly the comprehensiveness of NCD care, 41.2%); 41.2% on the availability of PHC services (particularly the competence of healthcare workers, 36.5%); 35.3% on population health management (particularly community-based services, 35.3%); 34.1% on facility organisation and management (particularly team-based care, 20.0%) and 31.8% on access (particularly digital technology, 23.5%). Most common strategies were task shifting and training to improve the comprehensiveness of NCD care through community-based services. Contextual factors related to inputs: infrastructure, equipment and medication, workforce (particularly community health workers), finances, health information systems and digital technology.

**Conclusion:**

Key strategies and contextual factors to improve PHC service delivery for NCDs in LMICs were identified. These strategies should combine with other strategies to strengthen the PHC system as a whole, while improving care for NCDs.

**Key messages**

- The Global Alliance for Chronic Diseases has invested in research that emphasises the importance of community-based services, task delegation within teams, training for better competency, digital technology for enhanced access to healthcare and comprehensive care for non-communicable diseases (NCDs).
- One of the most frequently used tactics to enhance the scope of NCD care provided via community-based services was through task shifting and training.
- The work outlines essential strategies and contextual factors that affect successful implementation. These must be integrated into a comprehensive approach to strengthening primary healthcare as a system.

## Introduction

 It has been well established that non-communicable diseases (NCD) are the leading cause of death worldwide, contributing to over 73% of annual deaths.^1^ Nearly 80% of the deaths in 2010 occurred in low and middle-income countries (LMIC),^2^ which have experienced rapid population ageing, urbanisation, rise in tobacco smoking and changes in diet and physical activity.^3^ Yet the primary healthcare (PHC) systems of LMICs, historically orientated to infectious diseases and maternal and child health, are not well designed to integrate NCD care. As a result, they are poorly prepared for the challenges of preventing and caring for people with cardiovascular diseases, diabetes, cancers and chronic respiratory diseases.^4^

PHC can promote healthy lifestyles, prevent disease, enable early diagnosis and improve treatment to reduce NCD-related morbidity and mortality.^5^ It can also be instrumental in rehabilitation that is needed to improve function and quality of life and palliative care.^6^ Existing literature has identified several potentially generalisable approaches to service delivery in PHC that are appropriate for NCD management. These include integration of services, team-based care, a greater focus on patient and community and harnessing information and communication technology.^4^ Given the size of the problem in LMICs, ongoing care for NCDs will need to be located within PHC services. In many countries, however, NCD care is far from fully integrated into PHC, and there are challenges more broadly in the health system of providing chronic as opposed to acute episodic care.^7^

The Global Alliance on Chronic Diseases (GACD) ‘brings together major international research funding agencies specifically to address the growing burden of NCDs in LMICs and vulnerable populations in high-income countries (HICs)’.^8^ The GACD has focused on how to implement proven interventions in diverse settings and ‘seeks to provide the evidence necessary to reduce inequalities in coverage and outcome and support evidence-based policymaking in building programmes to enhance public health’.^8^

The Primary Health Care Research Consortium (PHCRC) aimed to ‘support the development, conduct and dissemination of LMIC-led research; foster links between evidence and policy by addressing stakeholder priorities; and catalyse a global network of collaborating partners to increase PHC research capacity in LMICs’.^9^ The PHCRC was developed to identify gaps in knowledge and address priority global research questions on strengthening PHC in LMICs.^10^ The PHCRC approached the GACD to help address one of its priority research questions, namely, ‘what is the most effective and sustainable PHC service delivery model for the management of chronic diseases in a resource-constrained setting?’ The PHCRC believed that by looking across all the research projects funded within a specified time duration by the GACD that they could extract key lessons on PHC-focused service delivery models and strategies. The objectives of this study therefore were:

1. To identify key components of PHC service delivery models for NCDs in LMIC settings from the work funded by the GACD.
2. To identify what has been learnt by the GACD grantees on strategies to implement these components.
3. To describe the contextual factors that influence the implementation of these strategies.

## Methods

### Study design

This was a convergent mixed methods study, which combined both quantitative and qualitative data from the projects funded through the GACD.

### Setting

The GACD is an alliance of major public research funding agencies that was formed from its precursor, the Grand Challenges Global Partnership.^8^ The agencies within the GACD, fund implementation science research, with the aim of improving uptake and scale-up of well-evidenced approaches to prevention and control of chronic diseases (cardiovascular diseases, diabetes, certain cancers, lung diseases and mental ill-health). In 2010, the GACD’s funding agencies collaborated on the first unified funding call for implementation research projects to address the burden of hypertension in LMICs and indigenous settings. This was followed by calls for implementation research on type 2 diabetes mellitus, lung diseases and mental health in the years 2013, 2015 and 2016, respectively. In 2017–2018, the GACD funded projects to study scaling-up evidence-based interventions at the population level for the prevention or management of hypertension and/or diabetes. Many of these projects made interventions in PHC settings across different regions.

### Conceptual framework

**Figure 1:**
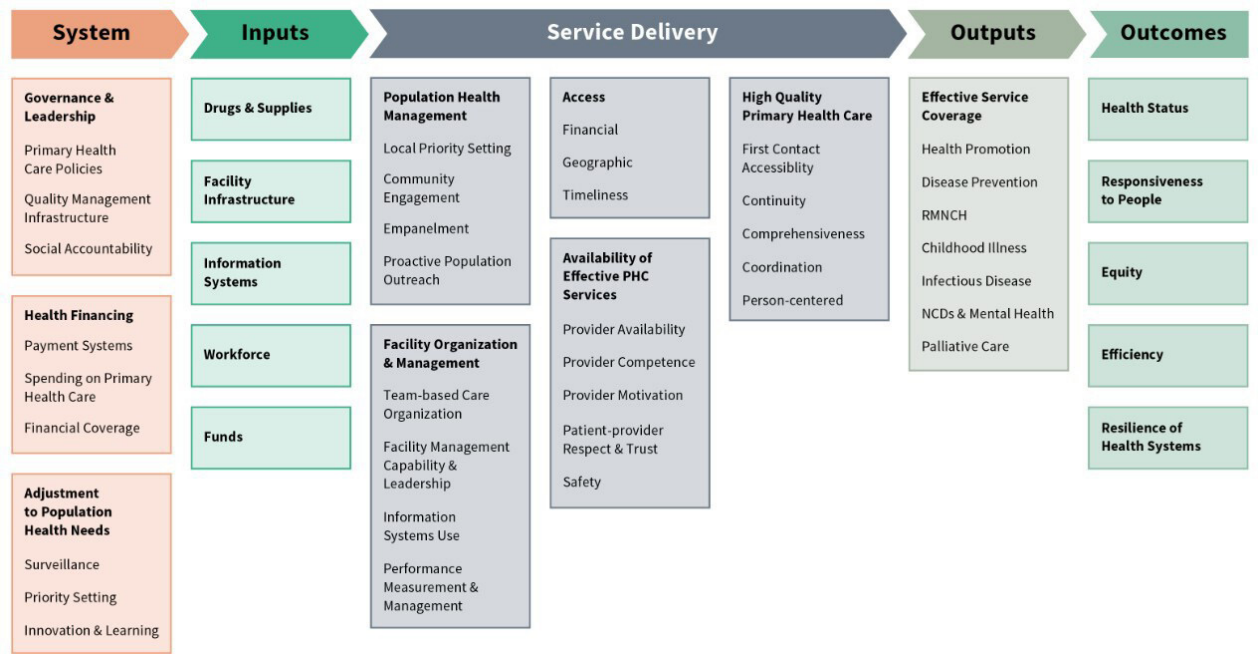
The PHCPI framework. NCDs, non-communicable diseases; PHCPI, Primary Health Care Performance Initiative; RMNCH, reproductive, maternal, neonatal and child health.^11^

The Primary Health Care Performance Initiative (PHCPI) framework (figure 1) was used as a conceptual framework.^11^ This framework was developed to help measure the key components of PHC and to inform and drive improvement. The framework has a logic model approach that moves from the system, to the inputs, to service delivery, and finally the outputs and outcomes. One of the focuses of the GACD projects was on implementing improvements in NCD service delivery. Service delivery in the framework is divided into 5 subdomains and 21 components that are further defined for the purpose of this study in table 1.

**Table 1:** Definitions used for allocation of studies to subdomains of service delivery in the PHCPI framework

Subdomains	Definitions
Population health management	Interventions that focused on the whole population at risk (in the catchment area of a PHC facility), rather than just the population attending the facility. Interventions could target community engagement, delineation or registration of the population served by the PHC facility (empanelment), processes for setting local health priorities, or proactive outreach to the whole population at risk. A component for community-based primary care services was added. This was for interventions that targeted part of the practice population (eg, people with diabetes), but in the community rather than the facility.
Facility organisation and management	This domain included interventions that helped the organisation or functioning of teams, developed leadership or managerial capability, improved information systems (including the patient’s medical record) or targeted systems for performance measurement and management.
Access	Interventions that targeted geographical access (overcoming the topography to access care), financial access (overcoming financial barriers to access care) or timeliness (that improved the linkage to care and time taken to access). Digital technology that took services or information to the people were also included as improving access.
Availability of effective PHC services	Interventions that improved the availability of health workers or professionals, their competence or motivation. This included improving the patient–provider relationship and safety of patients.
High-quality PHC	Interventions that improved the core functions of high-quality PHC. First contact accessibility refers to the accessibility, acceptability and utilisation of PHC when someone has a health need. Coordination of services could be between levels of care (sequential coordination when the patient is referred) or within levels of care (parallel coordination between members of the PHC team). Comprehensiveness refers to services across the life course, across the burden of disease and from health promotion to treatment and palliative care. Continuity refers to the availability of the patient’s information at each visit (informational continuity), systems for ensuring consistent and coherent ongoing care over multiple visits (management continuity) and building a relationship with a particular provider or small team (relational continuity). Person-centredness refers to the wholistic nature of the care and engaging in a bio-psycho-social approach to the person and their health problems.

PHCprimary health carePHCPIPrimary Health Care Performance Initiative

Components of the PHCPI framework have since been incorporated into the WHO’s new PHC measurement for impact framework.^12^ As the data were initially extracted using the PHCPI framework we have retained this as our conceptual framework.

### Selection of GACD projects and participants

The entire GACD database of funded projects till 2018 was reviewed to identify projects with a PHC focus. To be included, projects needed to be implementing in LMICs or in vulnerable communities within HICs. PHC implied services offered to people in a PHC facility or in the community served by that facility in social, learning, living or working spaces. Services could be offered by a wide variety of PHC providers, who could be in the public sector, private for-profit sector or private not-for-profit sector. Services could focus on any part of the spectrum from health promotion and disease prevention to rehabilitation and palliative care.

Projects were excluded if they focused on the general population and not a specific community or if the intervention was not delivered through or with PHC services (eg, changes to national legislation, hospital-based). Clinical trials of new treatments or diagnostic tests were also excluded.

These criteria were applied by one of the coauthors (ISK) to the GACD project summaries that were available on the website. Protocol papers or linked publications were also reviewed. Other coauthors (DP and MS) reviewed all excluded studies and any other studies where there was uncertainty.

### Extraction of data on selected projects

The following data were extracted from the GACD website and linked publications for all selected projects: project title, aim and objectives, study design and intervention that was implemented, country and income status (low, middle or high-income), and the NCD focus. Coauthors ISK, MS and RJ then used PHCPI subdomains to extract information on the study intervention. In each domain, the most important component was also identified.

### Primary qualitative data collection on selected projects

Sixteen projects were purposefully selected for key informant interviews to explore the issues in more depth during 2023. The selection was guided by the criteria that projects should cover all the service delivery subdomains, include a variety of NCDs and cover diverse regions. The final number of interviews was determined by saturation of data, when the last two interviews did not suggest any new themes.

An interview guide was developed to guide the semi-structured interviews with the principal investigator from the selected projects or with research team members, nominated by the principal investigator. The guide explored the model of care, the relationship of the intervention to the PHCPI framework domains, the implementation strategies, the barriers and enablers to implementation and the key findings or lessons with regards to implementation and the model of care.

### Data analysis

Quantitative categorical data on the characteristics of the projects were analysed descriptively and presented as frequencies and percentages. Qualitative data were captured in a verbatim transcript of the recording and checked for errors. The transcripts were then thematically analysed in NVivo software using the framework method.^13^

- Familiarisation: the researcher familiarised herself with the transcripts and noted key ideas and issues that could be developed into codes.
- Coding index: codes were developed, based on the first step and organised into categories. Categories were based on the PHCPI framework for the service delivery subdomains and components as well as the objectives of the study. Therefore, a deductive (based on the PHCPI framework) and inductive (identifying themes within this framework) approach was used.
- Coding: all transcripts were coded.
- Charting: reports were created, based on the categories, where all coded data were brought together in one report.
- Interpretation: reports were interpreted to identify key themes and subthemes.

### Integration of findings

As this was a convergent mixed methods study, we attempted to integrate the quantitative and qualitative findings in the results section around the objectives of the study. This was preferred over a sequential presentation of quantitative and then qualitative findings.

### Patient and public involvement statement

No patients or advisors were involved in the study.

## Results

Overall, 84 studies met the criteria for selection and their characteristics are shown in table 2. Research projects focused on diabetes (35.3%), hypertension (28.3%), mental health (37.6%), lung diseases (9.4%) and ischaemic heart disease (1.2%). Ten (11.9%) projects focused on hypertension and diabetes. Out of the 32 studies on mental health, 14 focused on mental health in general, 7 on depression or suicide, 4 on psychosis or schizophrenia, 4 on substance abuse, 2 on dementia and 1 on attention deficit and hyperactivity disorder. Out of the eight studies on lung disease, five focused on tobacco cessation or exposure and three on chronic obstructive pulmonary disease (one included asthma as well).

**Table 2:** NCDs and geographic locations of studies on PHC funded through the GACD

Characteristics (N=84)	n (%)
Disease focus	
Diabetes	20 (23.5)
Diabetes and hypertension	10 (11.8)
Hypertension	14 (16.5)
Lung disease	8 (9.4)
Ischaemic heart disease	1 (1.2)
Mental health	32 (37.6)
World region	
East Asia and Pacific	25 (29.4)
Sub-Saharan Africa	23 (27.1)
Latin America and Caribbean	18 (21.2)
South Asia	18 (21.2)
Europe and Central Asia	13 (15.3)
North America	4 (4.7)
Middle East and North Africa	1 (1.2)
Countries by income	
LMIC	63 (74.1)
HIC	10 (11.8)
LMIC and HIC	12 (14.1)

GACDGlobal Alliance on Chronic DiseasesHIChigh-income countryLMIClow or middle-income countryNCDsnon-communicable diseasesPHCprimary health care

As intended, the majority of studies were conducted in LMICs, although 25.9% included vulnerable groups from HICs. The largest regional foci were in East Asia/Pacific, particularly China, sub-Saharan Africa, Latin America/Caribbean and South Asia, particularly India. There was only one study from North Africa and the Middle East region.

Table 3 presents how the intended strategies mapped onto the key subdomains of PHC and the components.

**Table 3:** The focus of strategies in terms of the PHCPI framework

Subdomains and their components (N=85)	n (%)*
Population health management	30 (35.3)
Proactive community outreach	16 (18.8)
Community-based primary care	14 (16.5)
Community engagement	10 (11.8)
Local priority setting	1 (1.2)
Empanelment	0 (0.0)
Facility organisation and management	29 (34.1)
Team-based care	17 (20.0)
Information system	5 (5.9)
Performance management	4 (4.7)
Leadership and management capability	3 (3.5)
Access	27 (31.8)
Digital technology	20 (23.5)
Geographic	5 (5.9)
Financial	2 (2.4)
Timeliness	1 (1.2)
Availability of PHC services	35 (41.2)
Competence	31 (36.5)
Motivation	3 (3.5)
Availability	2 (2.4)
Patient-provider relationship	2 (2.4)
Safety	0 (0.0)
High-quality PHC	42 (49.4)
Comprehensiveness	35 (41.2)
Coordination	3 (3.5)
Continuity	2 (2.4)
Person-centredness	2 (2.4)
First contact access	0 (0.0)

* Projects could target more than one subdomain.

PHCprimary health carePHCPIPrimary Health Care Performance Initiative

Quality PHC was the most common subdomain to be targeted (49.4%), with almost all the studies focused on improving the comprehensiveness of NCD care (41.2%). For example, by adding aspects of care that were previously neglected, such as diagnosis of mental health disorders, patient education or disease prevention. Very few studies targeted continuity of care (2.4%), coordination of care (3.5%) or person-centredness (2.4%). No studies targeted first contact access to PHC facilities:

Comprehensiveness. I guess it depends how you define that. I mean part of the education that we provided to healthcare practitioners was around I guess the postpartum care for women who've had diabetes in pregnancy to ensure that they do have comprehensive checks across, we developed a resource called the key 5… It was more focused on preventive care, so they focused on women who had gestational diabetes. ……. They focused on prevention of complications and, you know, living healthily with diabetes (Project 1, Diabetes in pregnancy, Australia).

The second most common subdomain was the availability of effective PHC services (41.2%). Almost all of these studies targeted the competence of selected primary care providers (36.5%) through various forms of training or digital decision support.

We had trainings. Cognitive behavioural therapy (CBT) wasn't available or wasn't even included in the healthcare packages or in the education for even of a lot of psychiatrists or psychologists in their countries. So, in the beginning, together with one partner from the Netherlands, they developed a sort of short training on CBT. They went to the sites, did live training and competence building (Project 54, Mental Health, Central & Eastern Europe).

So, the ASHAs [Accredited Social Health Activists] certainly didn't know a lot about hypertension and part of the training was to improve their knowledge and to give them knowledge about risk factors in prevention (Principal investigator, project 19 (Hypertension, India).

In contrast, very few studies directly targeted the availability of providers (2.4%), the provider–patient relationship (2.4%), motivation of providers (3.5%) or patient safety (0%).

The third most common subdomain was population health management (35.3%). Many studies included interventions to do proactive outreach to the whole population to promote health, prevent disease or screen the population (18.8%), usually via community health workers (CHWs) or digital technology. Many studies also provided primary care for diagnosed diseases in the community (16.5%), usually via CHWs. A number of studies had community engagement and participation as a key aspect of the intervention (11.8%). No studies targeted local priority setting or empanelment:

It was a combination of things. Educating the population or the sample population about hypertension. It was just awareness knowing that they had hypertension. So screening was clearly important, and the intervention was focused really on the main target population to try and reduce blood pressure. So, it was about behaviour change mainly (Project 19, Hypertension, India).

In India, people already knew about hypertension and diabetes whereas there was no awareness in Indonesia when we implemented this App in 2016 and then we had to build on this program before the start of any intervention. So, I think that population levelness is key (Project 20, Cardiovascular disease, India)

The fourth most common subdomain was facility organisation and management (34.1%). Most of these studies targeted team-based care (20.0%) and this usually included task shifting to healthcare workers with lower levels of training, such as CHWs, and to other health professionals, such as pharmacists. Only 3.5% of studies targeted leadership, management capability, 5.9% the information system and 4.7% performance management:

So, they shifted some of the tasks to community health workers who got a broader scope of practice. That is how they somehow reached out to the community and managed to narrow the gap between what was happening at community level and what they could control from the facility. That part is quite important because it was stronger in the COPC [community orientated primary care] sites than in the non COPC sites, so already pointing to the to the impacts or the benefits of having the community health workers already in place as part of a community outreach team (Project 79, CVD, South Africa)

The least common subdomain targeted was access (31.8%). Most of these studies focused on various types of digital technology (23.5%) to communicate with or capacitate patients directly in the community, for example, using mHealth technology or social media. Only 5.9% of studies targeted other geographic barriers, 2.4% financial barriers, and 1.2% the timeliness of access:

We showed in the government that SMART health is useful for their people. Say, for example, now people got better access to medication, and they got free screenings. Previously to get access people would go to primary care, which is takes too long. And when they arrive at the primary healthcare, sometimes there is no medical doctor and so on and so forth. But with SMART health, the service is now going to them, which is very close to them (Project 76, CVD, Indonesia)

**Figure 2:**
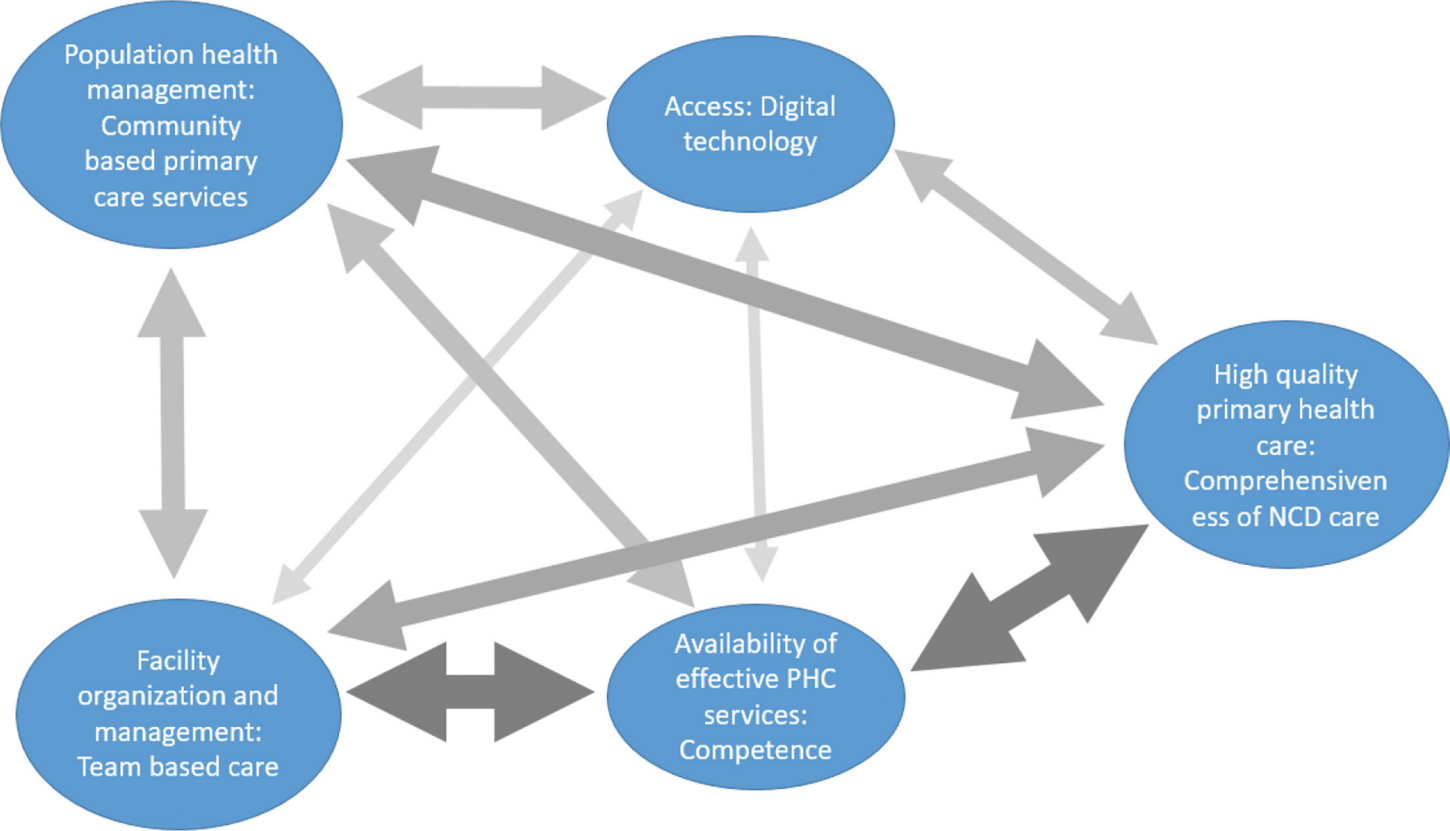
Engagement of multiple subdomains of the PHCPI in the GACD projects. The width and tone of the arrows are correlated to the number of projects that included both subdomains. GACD, Global Alliance on Chronic Diseases; NCDs, non-communicable diseases; PHCPI, Primary Health Care Performance Initiative.

Figure 2 shows the frequency with which more than one subdomain was targeted within the same studies. The most common shared subdomains were between facility organisation and management, availability of PHC services and high-quality PHC. For example, many studies engaged with task shifting and training to improve the comprehensiveness of care for NCDs, with a particular emphasis on community-based services. Another important inclusion of multiple subdomains was the use of digital technology to support community-based services and improve the comprehensiveness of NCD care.

### To identify what has been learnt by the GACD-funded studies on strategies to implement the interventions and important contextual factors

We extracted strategies and important contextual factors from the projects that were interviewed as shown in table 4. The lessons learnt are discussed below under two subheadings that focus on health system inputs and service delivery.

**Table 4:** Examples of strategies per PHCPI subdomain from the 16 project interviews and links to specific components and other subdomains

Strategies per subdomain	Main subdomain component targeted	Other subdomains linked to the project
Population health management		
Engaging with local elected and community leaders to support health activities	Community engagement	
Participatory discussions with community members on needs and gaps related to services for NCDs	Local priority setting	
Establishing community advisory boards to consult on service delivery for NCDs	Community engagement	
Screening services for NCDs provided by CHWs at household level	Pro-active population outreach	Access to services
Mass communication (offline) on NCDs and risk factors for example, posters, street theatre	Pro-active population outreach	
Using cultural activities or events to create awareness of NCDs and encourage uptake of health services	Pro-active population outreach	
Engaging community mobilisers to reach out to the population on NCDs	Pro-active population outreach	Access to services
Provide better transport for CHWs	Pro-active population outreach	Access to services
Establish support groups to improve self-management of NCDs	Community-based primary care services	High-quality PHC
Use of peers with lived experiences of NCDs to help provide services	Community-based primary care services	High-quality PHC
Community/home visits by service providers from the PHC facility	Community-based primary care services	High-quality PHC
Facility organisation and management		
Better supervision and support for CHWs from clinicians at the PHC facility using digital technology	Team-based care organisation	High-quality PHC
Improve medical record by software to record patients’ vital signs and status, monitor over time and assign tasks to service providers	Information systems	High-quality PHC
Software to track and inform patients about out-of-stock medications	Information systems	Access
Access		
Training pharmacists and medicine shop owners in screening for NCDs	Geographic access	
Messaging service to improve adherence, enable lifestyle change, modify risk factors, and provide remote consultations via telephone.	Geographic access	
Provide more integrated or new services closer to the community	Geographic access	
Improving the linkages between facility-based and community-based services to ensure timely care	Timeliness	High-quality PHC
Availability of effective services		
Advocacy with the health system to ensure availability of service providers at the primary care facilities	Provider availability	
Training for CHWs to provide community-based services online or on-site	Provider competence/motivation	
Mentoring and support to CHWs	Provider competence/motivation	
Digital tools to support guideline-based clinical decision-making by clinicians and health workers	Provider competence	
Financial compensation for CHWs to provide additional tasks	Provider motivation	
High-quality primary healthcare		
Engagement with family members to support self-management and lifestyle change via CHWs	Comprehensiveness	Population health management
Improved patient education and counselling for NCDs	Comprehensiveness	
Use of software by patients to monitor their indicators and support self-management	Comprehensiveness	
Software to improve coordination of community-based and facility-based services and ensure timely referral	Coordination	Facility organisation and management

CHWcommunity health workerNCDsnon-communicable diseasesPHCprimary health carePHCPIPrimary Health Care Performance Initiative

### Health system inputs

Strategies identified, targeted a number of inputs including workforce, infrastructure, equipment and medication, health information systems and digital technology. CHWs were widely added to the workforce and most community-based strategies made use of CHWs. Contextual factors included the willingness of CHWs to learn new competencies. However, there were many barriers to their effective performance, including lack of equipment and supplies to perform the expected tasks or inadequate training and supervision to be fully competent in using the equipment when it was available (eg, glucometers, sphygmomanometers):

As a part of the project, we developed an intervention component …. consisted of three main points. So, one is providing the technology platform for the ASHA [Accredited Social Health Activists] workers and the doctors. Second is the capacity development. So training, provision of user manuals, ensuring that the ASHA workers are able to diagnose to test blood pressure and diagnose blood glucose at the household level……. And the third component was kind of ensuring that there is a communication between the frontline health workers, the ANMs (Auxiliary Nurse Midwifes] and the doctors (Project 20, CVD, India)

Another challenge to this strategy was that facility-based workers might feel overburdened and unable to support CHWs. The facility-based workers were sometimes in short supply, and also poorly equipped and poorly supplied, especially with medication. Health services also lacked the mechanisms to recruit and pay peer supporters, which was a strategy tried by a number of studies, particularly in the context of mental health projects:

Without the proper infrastructure, there’s no way that your service delivery can work. So if there is no lab, if there are no medicines, and if the population has no money to buy medicines, you know it’s a long-term effect. It’s a long-term sort of thing. So you need to have inputs and you need to have proper systems (Project 20, Cardiovascular disease, India)

Strategies that involved CHWs were also limited in various contexts by inadequate or no remuneration, which appeared to effect motivation, attrition, the numbers of CHWs and reach. Good communication and coordination between community-based and facility-based services was also essential:

We have issues with the health workforce in terms of there being quite a lot of turnover. So often people are in positions for a short period of time before they move on to something else (Project 1, Diabetes in pregnancy, Australia)

Task sharing was sometimes poorly implemented, with lack of clearly defined new roles and responsibilities, and facility-based health professionals did not function as a truly multidisciplinary and integrated team. This resulted in a failure of the implementation of the strategy:

The nurses were quite overburdened. They didn't know what their role was, and the psychiatrist was basically the decision maker. So that is a barrier because it’s not clear how they can operate in this team? (Project 54, mental health, Central & Eastern Europe)

When allocating new tasks to healthcare workers (eg, supervising CHWs), it was important to check existing workload, capacity and workplace hierarchies. People resisted new tasks that stretched their capacity too far, or which required further support or approval from their managers. Teamwork was often dependent on managing the power dynamics between different types of providers to increase collaboration.

Training of service providers was a common strategy and needed to be based on assessment of their learning needs. In addition, hybrid (onsite and online) educational approaches were seen as useful. Some lessons emerged on pedagogy. Projects reported that training based on experiential learning that was regularly reinforced was a preferred approach. Some thought that group training of CHWs worked better than individualised training. Training of service providers should also include ‘soft skills’ such as communication (eg, empathic listening). Training of CHWs appeared to increase their capability and motivation. Other workforce-related strategies included advocacy for provider availability, and leveraging research on availability of needed skills and effective incentives for service providers.

Advocacy was also attempted as a strategy to improve infrastructure and supplies. PHC services should ensure availability of free or affordable medication and ensure sufficient understanding of the regimens to enable adherence.

In terms of strategies to improve the health information system, real-time availability of electronic patient data or records did not seem to contribute to better clinical decision-making and quality of care. Respondents reported that health information systems that included monitoring, reporting and accountability systems supported implementation.

Many strategies made use of digital technology. The design and development of new products were costly and automated systems difficult to set up. Where patients were the end users of such technology, it was important to repeatedly verify their correct phone numbers, to develop the technology with feedback from users, to use devices with which people were already familiar or very easy to use and to provide information about data security. Both patients and healthcare workers were apprehensive and hesitant about using new technology, especially when brought in from the outside. Sometimes, the electronic messaging was advocating services that did not actually exist. Nevertheless, information systems and the use of digital technology improved when the governance and managerial structures provided clear support, which was reinforced by policy:

The acceptance of an external software. It’s a very, very big problem. The security of that data or personal data…. And every clinic is more concerned about their data. But we always try to convince them that the data is private, that we have security measures, and the data is only for research and improved purposes. We don't have any commercial bias. We don't have any relationship with the pharmaceuticals for another industry. We only want to improve the quality of care in other sites, but it’s very difficult to get acceptance in some government scenarios (Project 10, Type 2 Diabetes Mellitus, Mexico))

### Service delivery

Strategies for strengthening health service delivery can be related to community engagement, multisectoral action and integrated PHC services. Community engagement needed to involve the recognised leadership, including religious leaders, to promote buy-in and uptake of services. Community engagement was often more informational, although it could improve health literacy and a desire to learn more. Community engagement could build on existing mechanisms and structures, particularly where there were prior community-based services and CHWs. Engagement should be flexible in terms of when it happened to maximise participation (not always during normal working hours). Involving community members in the design of interventions was likely to increase success and activities that also addressed the broader social determinants of health were likely to see more community participation. In some communities, gender segregated groups appeared to work better:

I think ideally in terms of the right people to be doing community engagement. They would hopefully be people who have already been working with the community for a period of time before the intervention so that there is some established relationship because for us certainly those relationships made a difference as to whether there was engagement in in any particular area (Project 1, Diabetes in pregnancy, Australia).

Strategies for multisectoral action emphasised the need for regular engagement and continuous collaboration with all stakeholders. Stakeholders included healthcare associations that could influence policy and governance structures. Engaging stakeholders from other sectors was necessary to address the social determinants of health and support lifestyle change.

Strategies to support integrated PHC services noted that facility-based health workers were more likely to support initiatives in the community if they were likely to reduce their own workload. Clearly articulating the evidence for new interventions was also likely to increase buy-in. Codesign of interventions with service providers and users was also likely to ensure relevance and feasibility. Other strategies included identifying ‘champions’ among system-level actors, who could spearhead new initiatives. Integrated primary care services at the community level were preferred, rather than vertically organised NCD services.

## Discussion

### Summary of key findings

We found that the research projects within the GACD focused on specific subdomains in the service delivery domain of the PHCPI framework. The highest focus was on population health management and specifically proactive population outreach to reduce risks of NCDs and improve knowledge as well as expand community-based primary care services for people with NCDs, usually involving CHWs. In facility management and organisation, the focus was on the composition of teams and task shifting to less trained and more affordable healthcare workers. Under access, there was strong use of digital technologies to improve utilisation or engage with people at a distance. In the subdomain of effective PHC services, another focus was on improving competence of the available healthcare workers through training and digital interventions to improve the comprehensiveness of services for NCDs. These focal points were often linked. For example, task shifting might be coupled with training of healthcare workers to improve their competence and the comprehensiveness of care for NCDs.

### Discussion of key findings

Most of the studies focused on diabetes and hypertension, with much less of a focus on cancer, chronic respiratory diseases, stroke and cardiovascular disease, as noted elsewhere.^14^ Although mental health problems were well represented. When looked at through a PHC system lens,^12^ projects within the GACD focused on issues that targeted NCDs more directly. While this focus was relevant and appropriate for improving the care of people with NCDs, it neglected important aspects of the PHC system and service delivery that were more cross-cutting. For example, how the local population was registered with or affiliated to a PHC service, whether NCD care was regarded as a priority by the local population, and how capable was the facility-level management and leadership. Similarly, few projects focused on accommodation of access, such as opening times, appointment systems or local transport as these would apply to all conditions and not just NCDs. Again, projects did not focus on the availability of healthcare workers, as this would be an issue for all conditions. Surprisingly, few projects focused on person-centredness in the consultation, improving continuity of care or coordination of care, all of which are vital for chronic care.

In addition, much of the work on improving quality of PHC services for NCDs focuses on clinical practice rather than policy or organisational levels.^14^ Even within clinical practice, there has been a focus in research studies on diagnosis and treatment, and relative neglect of issues related to health promotion, disease prevention, palliative care, self-management and health education.^15^ Health system inputs and the supply side of NCD care have been studied and found to be inadequate.^15^ Integration of NCD-related services into PHC also requires an enabling policy environment and governance mechanisms.^16^

There is, therefore, a potential weakness within the focus of studies, towards a more vertical programmatic thinking and away from a more horizontal systematic thinking. Such thinking is very common within health systems and funding agencies and can lead to inequity by disease.^17^ For example, at one point, the donated budget for HIV in Zambia far exceeded the entire national budget of the health system, for all diseases.^18^ We need to develop PHC as a system and nurture multiple cross-cutting elements that will improve care for all people and conditions. There have been calls for donor agencies to ensure that 30% of their funds go towards strengthening PHC as a system by 2030, rather than disease-related programmes.^17^

Many of the projects targeted improving population health management. We created a new component to differentiate between interventions that targeted the whole population at risk in the catchment area (eg, addressing risk factors in the population) versus interventions that shifted primary care for people with NCDs from the facility into the community (eg, adherence support for people with diabetes). The latter could be seen more as a strategy to shift tasks into the community and improve access, rather than population health management. The need to investigate the perspectives of users and communities and to tackle the underlying determinants of NCDs have been noted.^15^

CHWs were a popular element in many of the interventions. Many of the issues identified resonate with the literature on community-orientated primary care and barriers to the successful implementation of CHW teams.^19^ For example, key issues include providing the necessary remuneration, resources and equipment, supportive supervision, ongoing training, relationships with health professionals at the PHC facility, and ensuring acceptance by the community.

In PHC systems, the capability of facility level management and leadership is critical to the success of service delivery.^20^ This appears to be a neglected, but vital area, as ‘the fish rots from the head’.^21^ Leadership is responsible for the organisational culture, provision of inputs, success of teamwork, motivation, innovation and the expression of many other key values.^21^ Too often management at this level is more administrative, and in a ‘command and control’ style of leadership.^22^

Researchers were clearly keen on experimenting with digital technology as a way of improving access, because even people in poor communities have access to smart phones. We created a new component under access, to quantify the use of digital technology, a category that is also included in the new WHO framework.^12^ Interventions might focus on improving adherence via text messages, supporting lifestyle changes or modifying risk factors and providing remote consultations. Project learning appeared to resonate with the NASSS framework on ‘non-adoption and abandonment of technologies by individuals and the challenges to scale-up, spread and sustainability of such technologies in health and care organizations’.^23^ This framework emphasises the complexity of introducing new technologies into health systems and the frequency with which seemingly good ideas, fail to be sustained. Many of the contextual factors mentioned relate to the fourth domain in this framework, the adoption of new technology. This outlines that new technology may be resisted not just because of the need for new knowledge and skills, but because values or norms of practice are threatened by the new technology or place new burdens on patients for entering data or making decisions with the technology. Typical barriers include technological challenges, internet connectivity, poor computer literacy and lack of training.^24^

In many healthcare systems, it is the scarcity of healthcare workers that need to be addressed, and not just their competence or allotted tasks.^25^ Healthcare workers who are overburdened will often resist taking on new skills or tasks that increase their workload or slow down clinical care.^26^ Each health system must decide on what kind of multidisciplinary team is possible in PHC with the available resources.^27^ Almost always it is a balance between reach and coverage on the one hand (often involving less trained and inexpensive workers such as CHWs) and quality on the other hand (often requiring the presence of higher level expertise in the team, such as family doctors)—contributing to universal health coverage as one of the expected results.

The WHO now refer to the issues in the PHCPI quality domain as the core functions of primary care.^12^ All are important in delivering chronic care and not just comprehensiveness. Many projects focused on ensuring that care included all the elements, for example, patient education and counselling, health promotion or disease prevention. Given the importance of continuity to chronic care,^28^ it is surprising that this was not a focus. Such a focus could address both the availability of the patient medical record at each encounter (eg, electronic patient medical record) and the relational continuity of care (eg, building a relationship of trust with a particular provider or small team). Continuity also enables coordination of care, which can be parallel coordination (eg, between CHWs in the community and facility-based healthcare workers) or sequential coordination (eg, between PHC and the next level of expertise). Person-centredness is another cross-cutting function that implies a bio-psychosocial approach in the consultation and a responsiveness to the needs of users of the healthcare system.^29^ The care for NCDs will not achieve quality without attention to all the core functions.

### Strengths and limitations

The extraction of data depended on the information available on the GACD website and any publications. The GACD had a particular focus on certain diseases in the funding calls and did not cover all possible NCDs. Many projects were still busy implementing, collecting data, analysing and reporting. Particularly because the COVID-19 pandemic disrupted projects in the more recent waves of funding. The research team took care to clearly define the subdomains and components and to have a shared understanding of how to categorise projects. The qualitative interviews added more in depth understanding of the challenges to implementation in different contexts. Although a range of projects were interviewed and data saturation achieved, it is still possible that some key lessons could be missed. The projects come from all regions of the world, although the Middle East and North Africa had only one study. A paucity of studies from this region has been noted elsewhere,^14^ and it is our hope that the findings can be transferable to LMIC settings.

## Conclusions

The GACD research studies suggest that the models of PHC for NCDs should focus on proactive population outreach, community-based services, CHWs, task shifting within multidisciplinary teams, digital technology, training of PHC teams and interventions that improve the comprehensiveness of care for people with NCDs. At the same time, the mapping of these interventions to the PHCPI framework and a health systems perspective reveals areas that may be neglected, for example, empanelment of the population, facility management and leadership, access to care, availability of healthcare workers, continuity, coordination and person-centredness. In order to improve care for NCDs, the researchers within the GACD should ensure that they strengthen the whole PHC system and avoid the perspective of seeing NCDs as a vertical programme.

## Data Availability

Data are available upon request.
